# Management of Cervical Cancer in Pregnant Women: A Multi-Center Retrospective Study in China

**DOI:** 10.3389/fmed.2020.538815

**Published:** 2020-12-07

**Authors:** Mingzhu Li, Yun Zhao, Mingrong Qie, Youzhong Zhang, Longyu Li, Bei Lin, Ruixia Guo, Zhixue You, Ruifang An, Jun Liu, Zhijun Zhang, Hui Bi, Ying Hong, Shufang Chang, Guoli He, Keqin Hua, Qi Zhou, Qinping Liao, Yue Wang, Jianliu Wang, Xiaoping Li, Lihui Wei

**Affiliations:** ^1^Department of Obstetrics and Gynecology, Peking University People's Hospital, Beijing, China; ^2^Department of Obstetrics and Gynecology, West China Second Hospital of Sichuan University, Chengdu, China; ^3^Department of Obstetrics and Gynecology, Qilu Hospital of Shandong University, Shandong, China; ^4^Department of Obstetrics and Gynecology, Jiangxi Maternal and Child Health Hospital, Nan Chang, China; ^5^Department of Obstetrics and Gynecology, Shengjing Hospital of China Medical University, Shenyang, China; ^6^Department of Obstetrics and Gynecology, The First Affiliated Hospital of Zhengzhou University, Zhengzhou, China; ^7^Department of Obstetrics and Gynecology, The First Affiliated Hospital of Nanjing Medical University, Nanjing, China; ^8^Department of Obstetrics and Gynecology, The First Affiliated Hospital of Xi 'An Jiaotong University, Xi'an, China; ^9^Department of Obstetrics and Gynecology, Beijing Chaoyang Hospital, Capital Medical University, Beijing, China; ^10^Department of Obstetrics and Gynecology, Guizhou Provincial People's Hospital, Guiyang, China; ^11^Department of Obstetrics and Gynecology, Peking University First Hospital, Beijing, China; ^12^Department of Obstetrics and Gynecology, Nanjing Drum Tower Hospital, Nanjing, China; ^13^Department of Obstetrics and Gynecology, Second Affiliated Hospital of Chongqing Medical University, Chongqing, China; ^14^Department of Obstetrics and Gynecology, Hainan General Hospital, Hainan, China; ^15^Department of Obstetrics and Gynecology, The Obstetrics & Gynecology Hospital of Fudan University, Shanghai, China; ^16^Department of Gynecology Oncology, Chongqing University Cancer Hospital, Chongqing, China; ^17^Department of Obstetrics and Gynecology, Beijing Tsinghua Changgung Hospital, Beijing, China

**Keywords:** continuation, cervical cancer, pregnancy, neoadjuvant chemotherapy, termination

## Abstract

**Background:** This retrospective multi-center study aimed to describe the epidemiological characteristics, clinical features, and management of patients with cervical cancer in pregnancy (CCIP) and evaluate maternal and infant outcomes.

**Methods:** The data of patients with CCIP were retrospectively collected from those diagnosed and treated in 17 hospitals in 12 provinces in China between January 2009 and November 2017. The information retrieved included patients' age, clinical features of the tumor, medical management (during pregnancy or postpartum), obstetrical indicators (i.e., gestational age at diagnosis, delivery mode, and birth weight), and maternal and neonatal outcomes. Survival analyses were performed using Kaplan–Meier survival curves and log-rank tests that estimated the overall survival of patients.

**Results:** One-hundred and five women diagnosed with CCIP (median age = 35 years) were identified from ~45,600 cervical cancer patients (0.23%) and 525,000 pregnant women (0.020%). The median gestational age at cancer diagnosis was 20.0 weeks. The clinical-stage of 93.3% of the patients with CCIP was IB1, 81.9% visited the clinic because of vaginal bleeding during pregnancy, and 72.4% had not been screened for cervical cancer in more than 5 years. To analyze cancer treatments during pregnancy, patients were grouped into two groups, termination of pregnancy (TOP, *n* = 67) and continuation of pregnancy (COP, *n* = 38). Analyses suggested that the TOP group was more likely to be diagnosed at an earlier gestational stage than the COP group (14.8 vs. 30.8 weeks, *p* < 0.001). The unadjusted hazard ratio for the COP group's overall survival was 1.063 times that of the TOP group (95% confidence interval = 0.24, 4.71). There were no significant differences between the TOP and COP groups in maternal survival (*p* = 0.964). Thirty-three of the infants of patients with CCIP were healthy at the end of the follow-up period, with a median age of 18 ± 2.8 months.

**Conclusions:** Most patients with CCIP had not been screened for cervical cancer in over 5 years. The oncologic outcomes of the TOP and COP groups were similar. A platinum-based neoadjuvant chemotherapy regimen could be a favorable choice for the management of CCIP during the second and third trimesters of pregnancy.

## Introduction

China accounts for ~1/5th of the world's population. Cervical cancer is a common malignant tumor that seriously threatens the health of Chinese women. According to the National Cancer Report 2015, 98,900 cervical cancer cases were newly reported that year, causing 30,500 new deaths in China (1). The Chinese government has implemented several programs to control cervical cancer, including the National Cervical Cancer Screening Program in Rural Areas, which started in 2009. Due to variability in medical resources among regions, cervical cancer screening rates vary highly across regions, especially in rural and medically underserved areas (2, 3).

Cervical cancer in pregnancy (CCIP) is a rare event, which occurs in ~0.004–0.1% of pregnant and postpartum women (4, 5). The variation in the incidence of cervical cancer during pregnancy is likely to reflect the differences in underlying cervical cancer incidence across the population and screening programs (6). The management of CCIP is challenging and the rare nature of such conditions has resulted in a lack of reference data from randomized studies or large trials. Thus, the guidelines for the management of CCIP are currently based on limited data from a small number of cases and expert opinions (6–8). Since both maternal and fetal benefits need to be taken into consideration in the management of CCIP, it is imperative to provide an individualized approach and psychological support throughout pregnancy, and treatment decisions should be made by collaborative and multidisciplinary teams consisting of gynecologic oncologists, obstetricians, pathologists, and neonatologists (9).

In recent years, treatment for CCIP has gradually shifted from aggressive therapies to more pregnancy-preservative management, particularly for patients in the early stages of cervical cancer within the second or third gestational trimester (10). Fertility-preservative options that include radical or simple trachelectomy with or without neoadjuvant chemotherapy (NACT) have been successfully applied in cervical cancer patients (11). Surgery may be proposed as the primary treatment for early-stage cervical cancer (10). In contrast, NACT is an optional treatment for patients with advanced-stages of cervical cancer, which may postpone the definitive local treatment until term or after delivery (10).

Given the limited data on maternal and fetal prognosis (i.e., continuation vs. termination of pregnancy), our study aimed to contribute to clinical evidence by assessing the clinical characteristics, management, and prognosis of cervical cancer in pregnant women with different gestational age (GA) and compare the subsequent outcomes of termination of pregnancy (TOP) and continuation of pregnancy (COP).

## Materials and Methods

This is a hospital-based retrospective descriptive study, in which patients who were diagnosed with CCIP from January 2009 to November 2017 (data retrieving period) were selected. Data on patients were collected from their medical records, which were kept in the archives of 17 hospitals located in 12 cities. These hospitals included the Peking University People's Hospital, Peking University First Hospital, Beijing Chaoyang Hospital, Capital Medical University and Beijing Tsinghua Changgung Hospital in Beijing; The Obstetrics & Gynecology Hospital of Fudan University in Shanghai; Qilu Hospital of Shandong University in Jinan; West China Second University Hospital of Sichuan University in Chengdu; The First Affiliated Hospital of Zhengzhou University in Zhengzhou; Jiangxi Maternal and Child Health Hospital in Nanchang; Guizhou Provincial People's Hospital in Guiyang; The First Affiliated Hospital of Xi'an Jiaotong University in Xi'an; The First Affiliated Hospital of Nanjing Medical University and Nanjing Drum Tower Hospital in Nanjing; The Second Affiliated Hospital of Chongqing Medical University and Chongqing University Cancer Hospital in Chongqing; ShengJing Hospital of China Medical University in Shenyang; and Hainan General Hospital in Haikou.

The study was approved by the lead hospital's (i.e., Peking University People's Hospital's) Institutional Review Board (PKUPH IRB, 2018PHB230-01), and all other 16 hospitals provided their IRBs' comments and agreed to follow the terms laid out by the lead hospital's IRB. Prior to their treatment, the enrolled patients' treatments were performed according to protocols that were decided by multidisciplinary teams, and patients provided informed consent, permitting their medical records (from diagnosis to follow-up) to be used for further analysis. As a result, the IRB approved the retrospective study without the need to obtain further written informed consent from patients. However, we still prepared an informed consent form for patients to sign by mail in case, (1) any patient requested to be removed from the study, and (2), in cases when an informed consent form signed by a patient from any subsite in the treatment stage did not clearly state that they permitted their data to be used for further research.

Patients were included in the analyses if they were diagnosed with CCIP during gestation, according to the FIGO (International Federation of Gynecology and Obstetrics) 2009 instructions for cervical cancer staging (12) and World Health Organization (WHO) criteria for histopathological diagnosis of cervical cancer, and had complete records (i.e., data) from diagnosis to follow-up.

The retrospective analysis was performed on the following parameters: each patient's basic information (i.e., age and gestational age, GA at diagnosis), tumor features (i.e., FIGO staging and histopathology), cancer management (during pregnancy or postpartum), patient response to treatment (as measured by results from clinical examinations and magnetic resonance imaging, MRI, or ultrasonography), obstetrical indicators (i.e., obstetric history, mode and GA of delivery, birth weight, 5-min Apgar scores, and neonatal outcome), and maternal outcome.

For analyses, patients were categorized into the TOP group if fetal preservation was abandoned and the COP group if the fetus was kept according to the patient's wishes. Treatment options encompassed by COP included a (1) surgery sub-group: surgical treatments including conization/radical trachelectomy ± lymphadenectomy, (2) NACT sub-group: NACT administration during pregnancy, and (3) post-delivery treatment (PDT) sub-group: regular follow-up for the tumor without definitive treatment until delivery. Termination of pregnancy refers to feticide treatment options such as hysterectomy or chemoradiotherapy with the fetus *in utero* or after a previous evacuation and are usually performed during the first or second trimesters. To analyze the overall survival, deaths from any cause by March 12, 2018 (the endpoint of the follow-up) were recorded. Patients who did not experience cancer recurrence or were alive at the end of the follow-up were censored at the last known date before or at the end of follow-up. The physical and cognitive development statuses of the children of the COP group were acquired during the follow-up.

### Statistical Analysis

Descriptive statistics were calculated for tumor features and obstetrical indicators. Categorical data were summarized using frequencies and percentages, and continuous data were summarized using means and standard deviations (SD) for normally distributed data and medians and ranges for non-normally distributed data. Clinical characteristics were compared between TOP and COP groups using 95% confidence intervals (95% CIs) for differences in means or proportions, as appropriate. The survival rate was compared using the Kaplan-Meier method, a survival curve was drawn, and log-rank tests were used to assess differences. SPSS 16.0 software (SPSS Inc., Chicago, IL, USA) was used for data processing and analysis. *P* < 0.05 was considered to indicate a statistically significant difference.

## Results

### Patient Characteristics at Diagnosis

A total of 105 women diagnosed with CCIP were identified from 45,600 patients with invasive cervical cancer (0.23%, 105/45,600). This represented 0.020% of the 525,000 pregnant women (105/525,000) who presented at the site hospitals during the same period. The median age at diagnosis of patients with CCIP was 35.0 ± 5.3 years (17–45 years). Thirty-eight (36.2%) of those patients were diagnosed in the first trimester of gestation, 33.3% in the second, and 30.5% in the third. Ninety-eight (93.3%) of patients with CCIP were histologically staged at level IB1 and above at the primary diagnosis. Squamous carcinoma (SCC) and adenocarcinoma (AC) accounted for 94.3% (99/105) of all the histology. Pregnancy was terminated in 63.8% (67/105) of patients (the TOP group), while pregnancy was continued in 36.2% (38/105) of the patients (the COP group). The median GA at diagnosis in the TOP group was 14.8 weeks (5–31weeks) and 30.8 weeks (6–41 weeks) in the COP group (*P* < 0.01).

Abnormal bleeding during pregnancy was the main symptom in 85.7% (90/105) of the patients. Notably, only five (4.76%) of the 105 included patients were screened for cervical cancer within the 5 years that preceded the diagnosis, and 76 (72.4%) of the patients had not been screened for cervical cancer for over 5 years at the time of diagnosis. Among the 24 (22.9%) patients screened during or before their pregnancies with cytology and/or HPV testing, 17 were tested due to cervical cancer-related symptoms, and seven were screened with cytology without showing any carcinoma-related abnormalities in their medical histories or physical examinations. Of the seven patients screened with cytology, four had ASC-US cytology, and three had HSIL cytology. They were diagnosed with CCIP due to abnormal biopsies. Except for the differences in stage IB2 and GA at diagnosis, there were no significant differences between the TOP and COP groups in patients' age, gravidity, parity, FIGO staging, and tumor size (Table 1).

**Table 1:** Descriptive statistics of demographic and tumor characteristics (*n* = 105).

	**Total (*n* = 105)**	**TOP (*n* = 67)**	**COP (*n* = 38)**	**Difference in means (95% CI)**
Age (yrs), mean (SD), range	35.0 (5.3), 17–45	34.2 (5.1), 17–44	32.3 (5.6), 23–45	1.8 (−0.2 to 4.0)
Gravida, mean (SD), range	3.6 (1.7), 1–9	3.8 (1.6), 1–8	3.2 (1.9), 1–9	0.6 (−0.4 to 1.3)
Para, mean (SD), range	1.3 (1.2), 0–7	1.4 (1.0), 0–5	1.3 (1.5), 0–7	0.1 (−0.3 to 0.6)
GA at diagnosis (wks), mean (SD), range	20.0 (11.1), 5–41	14.8 (7.7), 5–31	30.8 (8.5), 6–41	−16.0 (−19.3 to −12.8)^*^
**Method of disease detection**, ***n*** **(%)**				
Bleeding	90 (85.7)	57 (85.1)	33 (86.8)	−1.8 (−14.6 to 13.9)
Physical examination	8 (7.6)	4 (6.0)	4 (10.5)	−4.6 (−18.6 to 6.0)
Abnormal cervical cancer screening	7 (6.7)	6 (8.9)	1 (2.6)	6.3 (−5.6 to 15.8)
**FIGO Stage**, ***n*** **(%)**				
IA	7 (6.7)	3 (4.5)	4 (10.5)	−6.1 (−20.0 to 4.1)
IB1	30 (28.6)	19 (28.4)	11 (28.9)	−0.6 (−19.0 to 16.2)
IB2	33 (31.4)	26 (38.8)	7 (18.4)	20.4 (1.9 to 35.5)^*^
II	28 (26.6)	17 (25.4)	11 (28.9)	−3.6 (−21.7 to 13.1)
III–IV	7 (6.7)	2 (3.0)	5 (13.2)	−10.2 (−24.5 to 0.2)
**Pathological type**, ***n*** **(%)**				
Squamous cell carcinoma	83 (79.1)	53 (79.1)	30 (78.9)	0.16 (−14.8 to 17.4)
Adenocarcinoma	16 (15.2)	10 (14.9)	6 (15.8)	−0.9 (−16.9 to 12.5)
Other type^†^	6 (5.7)	4 (6.0)	2 (5.3)	0.7 (−11.8 to 9.9)
**Tumor size**, ***n*** **(%)**				
≤4 cm	46 (43.8)	30 (44.8)	16 (42.1)	2.7 (−16.7 to 21.2)
>4 cm	59 (56.2)	37 (55.2)	22 (57.9)	−2.7 (−21.2 to 16.7)

*TOP, termination of pregnancy; COP, continuation of pregnancy; GA, gestational age; yrs, years; wks, weeks*.

* *P < 0.01*.

† *Three cases of small cell carcinoma, one case of poorly differentiated carcinoma, one case of cervical sarcoma, and one case of neuroendocrine carcinoma*.

### Management During Pregnancy

In the COP group, cervical surgeries were only performed in three patients (surgery sub-group) who were diagnosed early, at an average of 18 weeks of GA and staged ≤ Ib2 (Table 2). Of the three patients, one patient with a stage IA2 tumor underwent conization at 12 weeks GA, and two patients with stage IB1 tumors underwent radical trachelectomy at 26 and 18.6 weeks of gestation, respectively. Among the two patients with stage IB1 cancer, the one who underwent radical trachelectomy at 26 weeks of GA also underwent lymphadenectomy. The other patient did not undergo lymphadenectomy but was treated with concurrent chemoradiotherapy (CCRT) after delivery, experienced cancer recurrence, and died within the 5-year follow-up. Platinum-based neoadjuvant chemotherapy was administered to 10 (26.3%) pregnant patients (the NACT sub-group) during the second or third trimester, at 26 (range 19–32) weeks of gestation on average. Eighty percent (8/10) of those patients had stage IB2 tumors ≥ 4 cm in size. Eighty percent (8/10) of the tumors in those patients were also classified as squamous cell carcinoma (Table 2). Cisplatin combination therapy was administered to all ten patients, of which eight received cisplatin plus paclitaxel, one received cisplatin plus vincristine and bleomycin, and one received cisplatin plus bleomycin and etoposide. On average, 3.5 cycles of chemotherapy were administered during pregnancy in dosages similar to those for non-pregnant patients, with adjustments made for patients' actual weight, height, and glomerular function. Complete responses to NACT administration were achieved by 20% (2/10) of the patients, while partial responses were achieved by 30% (3/10) of the patients. The lesions of 30% (3/10) of patients stabilized, and 20% (2/10) of patients had no observed sensitive response to chemotherapy, and their lesions progressed after NACT. These final two patients included a patient diagnosed with small-cell carcinoma and a patient with stage IIB cancer.

**Table 2:** Characteristics of patients in the COP group, stratified by management modality (*n* = 38).

	**Surgery sub-group (*n* = 3)**	**NACT sub-group (*n* = 10)**	**PDT sub-group (*n* = 25)**
Age (years)	35 (25-36)	33 (23-37)	34 (23-45)
GA at diagnosis (weeks)	18(6-25)	26 (19-32)	37 (9-41)
**FIGO Stage**, ***n*** **(%)**			
≤IB1	3 (20)	2 (13.3)	10 (66.7)
>IB1	0 (0)	8 (34.8)	15 (65.2)
**Pathological type**, ***n*** **(%)**			
Squamous cell carcinoma	2 (6.7)	8 (26.7)	20 (66.7)
Non–squamous cell carcinoma	1 (12.5)	2 (25)	5 (62.5)
**Tumor size**, ***n*** **(%)**			
≤4 cm	3 (18.8)	2 (12.5)	11 (68.8)
<4 cm	0 (0)	8 (36.4)	14 (63.6)
GA at delivery (weeks)	36 (34–38)	34 (30–35)	37 (28–41)
Delivery weight (grams)	3,290 (1,700–3,320)	2,352 (1,350–2,610)	2,980 (1,315–4,050)
**Mother's outcome**, ***n*** **(%)**			
DOD	1 (33.3)	0 (0)	2 (66.7)
NED	2 (6.5)	10 (32.3)	19 (61.3)^*^

*DOD, dead of disease; NED, no evidence of disease; ND, not determined; GA, gestational age; NACT, neoadjuvant chemotherapy; PDT, post-delivery treatment*.

* *Another four cases were lost to follow-up*.

Post-delivery treatment occurred in 65.8% (25/38) of patients (the PDT sub-group). Among them, eight had stage IB1, eight had stage II, two had stage IA, three had stage IB2, and four had stage IIIb cancer. Twenty-three of the 25 (92%) patients were diagnosed in the third trimester, at an average GA of 37 weeks (9–41 weeks) weeks, while the other two patients were diagnosed at 26 weeks and nine weeks of gestation, respectively.

Of the 38 patients in the COP group, a cesarean section was performed on 97.4% (37/38), and only one had a vaginal birth. Twenty-nine (76.3%) of the patients had premature births, while the other 9 (23.7%) had term deliveries with a mean GA of 35.0 ± 3.2 weeks. There were no significant differences among the three patient management subgroups (i.e., surgery, NATC, and PDT sub-groups) in GA at delivery (Table 2).

The treatments that patients in the COP groups underwent included:

- cesarean delivery combined with radical hysterectomy in three patients,
- post-delivery radical hysterectomy plus chemotherapy in 11 patients,
- post-delivery radical hysterectomy plus concurrent chemoradiotherapy (CCRT) in eight patients,
- post-delivery hysterectomy in three patients,
- post-delivery CCRT in four patients, and
- no treatment or unclear treatment in nine patients.

The median GA of the 67 patients in the TOP group at delivery was 15.2 ± 8.3 weeks. Among the 36 patients diagnosed in the first trimester, radical hysterectomy was performed on 18, abortion followed by radical hysterectomy on 13, abortion followed by CCRT on two, and details of the post-abortion treatment were not available for three patients. Among the 25 patients diagnosed in the second trimester, radical hysterectomy was performed on five, hysterotomy before radical hysterectomy on 13, and abortion followed by radical hysterectomy on seven patients. Radical hysterectomy following hysterotomy was performed on all six patients who were diagnosed in the third trimester. Post-operative adjuvant therapy was given according to the appearance of clinical high-risk factors.

### Patient Outcomes

**Figure 1:**
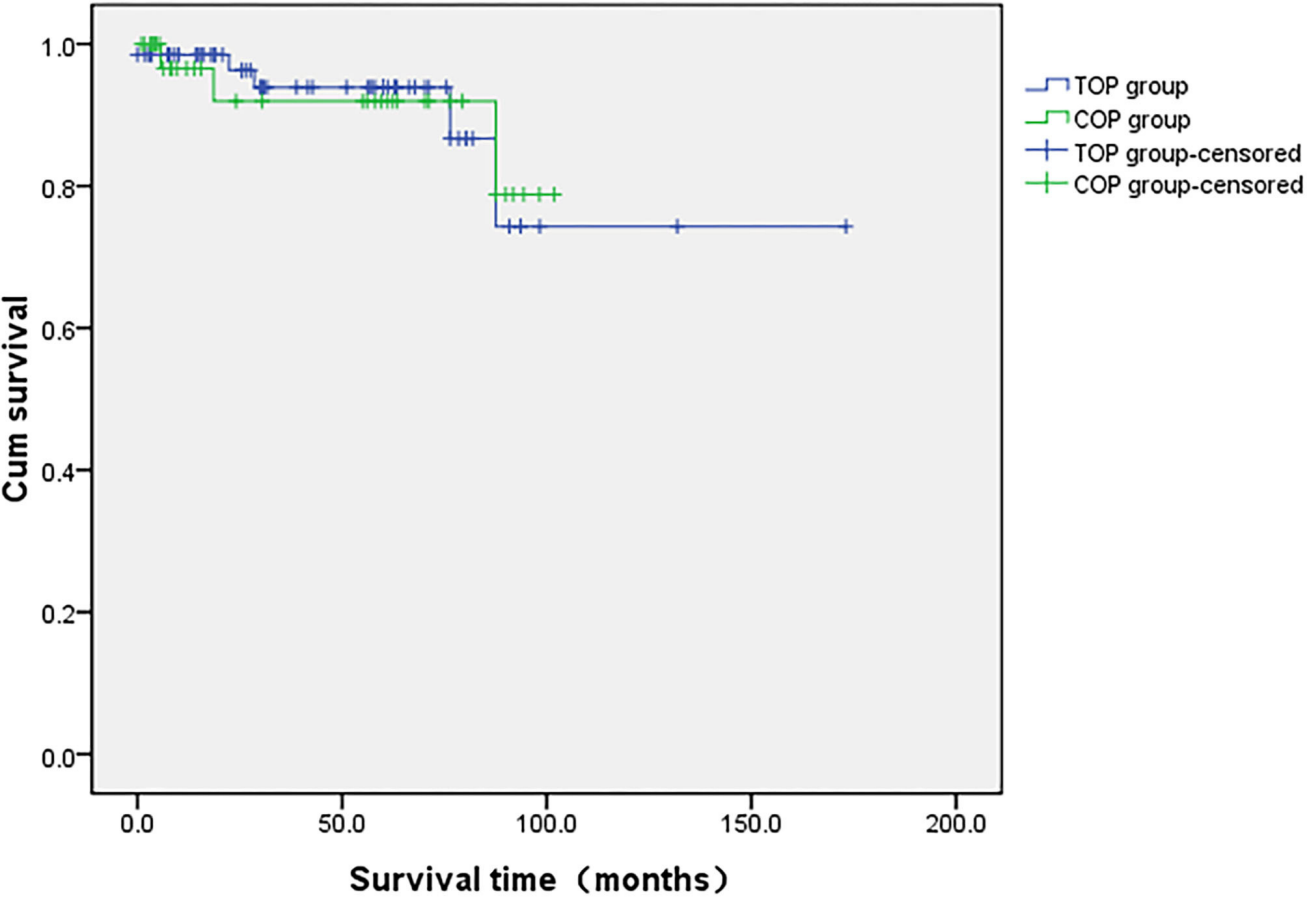
Comparison of overall survival in the COP and TOP groups.

The median follow-up time for the 105 patients was 61 ± 6 months (1-173). There were no significant differences in follow-up time between the COP and TOP groups (40 vs. 45 months, *p* = 0.532). During follow-up, 11 patients (10.5%) experienced a relapse in cancer (six in the TOP and five in the COP groups), eight (7.6%, five in the TOP and three in the COP groups) died of tumor progression, and 21 patients (20%, six in the COP and 15 in the TOP groups) were lost to follow-up. None of the COP patients who were administered NACT showed any evidence of disease recurrence and death. The unadjusted hazard ratio between the COP and TOP groups was 1.063 for overall survival (95% CI = 0.24, 4.71). Figure 1 displays the Kaplan–Meier curves for differences in survival since the diagnosis of cervical cancer between the COP and TOP groups (*p* = 0.964).

### Fetal Outcomes

In terms of obstetric outcomes, data on fetal outcomes at birth were collected and analyzed from 38 patients with CCIP. The median weight of newborns from the COP group was 2,554 ± 760 g (1,315–4,050 g). Six of the 38 (15.8%) newborns were in the lower 10th percentile of weight as compared with the normal weight of newborns at the same GA. This is typically identified as small for gestational age. With the exception of four newborns for whom information was unavailable, 32 of the newborns had Apgar scores ≥7 at birth, with only two having an Apgar score below seven. Thirty-three children who completed follow-up visits with full records, during an average of 18 ± 2.8 months (0.7–84 months) of follow-up, showed normal physical and cognitive development. Details of the 38 COP cases are shown in Table 3.

**Table 3:** Perinatal and pregnancy outcomes of COP cases (*n* = 38).

**No**	**Maternal age (years)**	**GA at diagnosis (weeks)**	**Stage**	**Pathology**	**GA at delivery (weeks)**	**Delivery mode**	**Delivery weight (grams)**	**Apgar score**	**Age at last follow-up**
1	36	34^+5^	IIIB	SC	35	CS	1,700	8–8–8	6Y
2	29	34^+2^	IB1	SC	35	CS	2,380	9–9–10	6Y
3	35	27^+4^	IB2	SC	31^+5^	CS	1,350	9–9–10	3M
4	36	25^+3^	IB1	SC	36^+3^	CS	3,320	9–9–10	2M
5	45	41^+1^	IIB	SC	41^+2^	CS	3,400	9–9–10	ND
6	38	40	IB2	AA	40^+2^	CS	ND	9–9–9	5Y
7	32	31	IIIB	SC	31^+4^	VD	1,600	10–10–10	5Y
8	24	38	IIB	SC	38	CS	2,500	ND	3Y
9	23	26	IB1	AA	28	CS	ND	ND	2Y
10	28	37^+1^	IIIB	SC	37^+1^	CS	2,400	10–10–10	10M
11	38	41	IB2	SC	41	CS	4,050	9–9–10	8M
12	35	36	IIA2	SC	36	CS	3,000	9–9–9	6M
13	34	28^+4^	IB1	SC	34	CS	1,315	9–8–8	4Y
14	27	20^+3^	IB2	SC	35	CS	2,460	10–10–10	23M
15	23	22^+5^	IIB	SC	35	CS	2,170	10–10–10	18M
16	34	32	IB2	SC	34	CS	ND	ND	1M
17	37	28^+2^	IIB	AA	30^+2^	CS	ND	ND	3M
18	32	26^+5^	IVB	SCLC	32^+5^	CS	2,500	9–9–9	7M
19	40	31^+6^	IA1	SC	35	CS	2,980	9–9–9	7M
20	29	38^+3^	IB1	SC	38	CS	3,850	10–10–10	7Y
21	40	37^+2^	IIA2	SC	37^+2^	CS	3,560	10–10–10	ND
22	27	35	IB1	SC	35	CS	2,060	9–10–10	ND
23	34	40	IIA1	SC	40	CS	3,320	10–10–10	ND
24	32	38^+4^	IA1	AA	38^+4^	CS	3,020	10–10–10	1M
25	29	34^+6^	IIIB	SC	34^+6^	CS	1,700	4–6–6	3M
26	25	18	IB1	AA	34^+2^	CS	1,700	9–9–9	4Y
27	24	34^+2^	IIB	SC	35^+1^	CS	2,480	10–10–10	7Y
28	26	31^+4^	IA1	SC	34^+5^	CS	2,330	9–10–10	3Y
29	40	37^+2^	IB1	SC	37^+5^	CS	3,420	10–10–10	7Y
30	26	38^+4^	IB1	SC	40	CS	3,020	10–10–10	2Y
31	34	9	IIB	SC	32^+5^	CS	1,700	10–10–10	30D
32	26	32	IIa2	SCLC	32^+4^	CS	1,750	6–8–8	ND
33	37	39^+2^	Ib2	AA	39^+2^	CS	3,000	10–10–10	2.5Y
34	32	19^+3^	IIB	SC	32^+3^	CS	1,900	8–10–10	20D
35	38	39^+6^	Ib1	SC	39^+6^	CS	3,600	10–10–10	4.5M
36	33	24^+4^	Ib2	SC	35^+3^	CS	2,610	9–10–10	8M
37	35	6	IA2	SC	38^+3^	CS	3,290	10–10–10	1Y
38	35	26^+6^	Ib1_	SC	34^+2^	CS	2,375	9–10–10	6Y

*SC, squamous cell carcinoma; AC, adenocarcinoma; SCLC, small cell carcinoma; CS, cesarean section; VD, vaginal delivery; ND, not determined; Y, year; Age: M, month; D, day*.

## Discussion

### Epidemiology of Cervical Cancer in Pregnancy in China

Due to a lack of specific data from randomized trials on the prevalence of CCIP in China, our study retrospectively assessed available records and determined the incidence of CCIP in China to be 0.020%, similar to what has been reported previously (13). The 35 years old median age at CCIP diagnosis suggests that childbearing at an older age might account for increased CCIP occurrence. It is worth mentioning that more than 90% of the CCIP cases were at least stage IB1 at the time of diagnosis. Squamous cell carcinoma was the most common subtype of CCIP and, therefore, characterized the disease's clinical and epidemiological picture (4). Vaginal bleeding was the main complaint, which might often be mistaken for potential miscarriage when the patient visited the clinic in early pregnancy or be misdiagnosed as premature labor or placenta previa when the patient visited the clinic in mid or late pregnancy. Surprisingly, 72.4% of the patients with CCIP included in our study had not been screened for cervical cancer within the 5 years that preceded diagnosis. This indicates that the HPV vaccine and cervical cancer screening are not enough in monitoring and preventing this type of cancer in China (14). Given this, the Chinese Society for Colposcopy and Cervical Pathology (CSCCP) has recommended cervical cancer prevention via regular screening and early detection should be integrated into public education about women's health (15).

### Management of CCIP Patients in China

There is not enough evidence to indicate the optimal treatment for CCIP patients. However, guidelines for the optional treatment of cervical cancer in pregnant patients have recently been published, which include expert recommendations based on limited data on cancer treatment efficacy during pregnancy (6, 7). Treatment decisions must consider many factors, including GA at diagnosis and patients' preferences for pregnancy outcomes. When the continuation of the pregnancy is not the purpose, management is similar to non-pregnant women. In our study, 91% (61/67) of the TOP patients terminated their pregnancies during the first or second gestation trimesters, and 9% (6/67) were in the third trimester when the potential exists to continue a pregnancy by taking postponed treatment. This indicates that knowledge of active management using surgery or NACT should be strengthened for options aimed at the continuation of pregnancy.

When a patient with CCIP chooses to preserve the pregnancy, several surgery protocols exist for the early stages of the disease (i.e., stage IA1–IB2), such as large conization or simple trachelectomy, which is gaining support (16, 17). Radical trachelectomy was a recommended option for young patients with early invasive uterine cervical cancer who decided to preserve their fertility. Some cases of radical trachelectomy have revealed that this procedure was challenging for gynecologic oncologists and obstetricians because of its operative radicality and the extremely high risk of complications (e.g., fetal loss, excessive bleeding, and prolonged surgery). Thus, it is not commonly recommended (6). In our study, radical trachelectomy was performed in two patients whose gestational weeks of delivery were 36^+3^ and 34^+2^, respectively. Of those two patients, one suffered from cancer recurrence, suggesting the importance of pelvic lymphadenectomy.

In patients with more advanced stages (i.e., IB2-IIA) of cancer, NACT is an alternative management option commonly administered during pregnancy (6, 10, 18). Proper application of NACT can stabilize the tumor, control the disease, prevent the tumor from dissemination, and postpone unanticipated delivery (19). There is growing evidence on maternal safety and satisfied obstetrical outcomes with NACT administration during pregnancy (9). A NACT regimen comprised of paclitaxel plus cisplatin may be a proper approach for patients with CCIP (20). In our study, two cases with larger volume tumors received NACT and showed good chemotherapeutic reactivity. However, two patients did not respond well to chemotherapy, of which one responded to CCRT and intra-arterial chemotherapy after delivery (21). This suggests that for patients who choose to continue the pregnancy, postponing the treatment until the second trimester (when diagnosed during early pregnancy) or using NACT in the second and third trimester could be considered.

### Maternal-Fetal Prognosis of Cervical Cancer in Pregnancy in China

Some literature has reported that CCIP has a poorer prognosis than cervical cancer in the general population of women due to its biological behavior. As a result, the proposed treatment protocols for CCIP were more active than those for more common cervical cancer (22). Increasingly, the literature has reported that pregnancy does not adversely affect the survival and prognosis of women with invasive cervical cancer (23–25). It is worth noting that our study did not find a difference in maternal survival between patients who terminated pregnancy and those who continued pregnancy. On the other hand, 33/38 children delivered to patients with CCIP were in good health and had no physical or intellectual disability at the end of follow-up. However, the effects of NACT administration on the fetuses of patients with CCIP and the development of long-term complications in children requires further monitoring and research (26).

**Figure 2:**
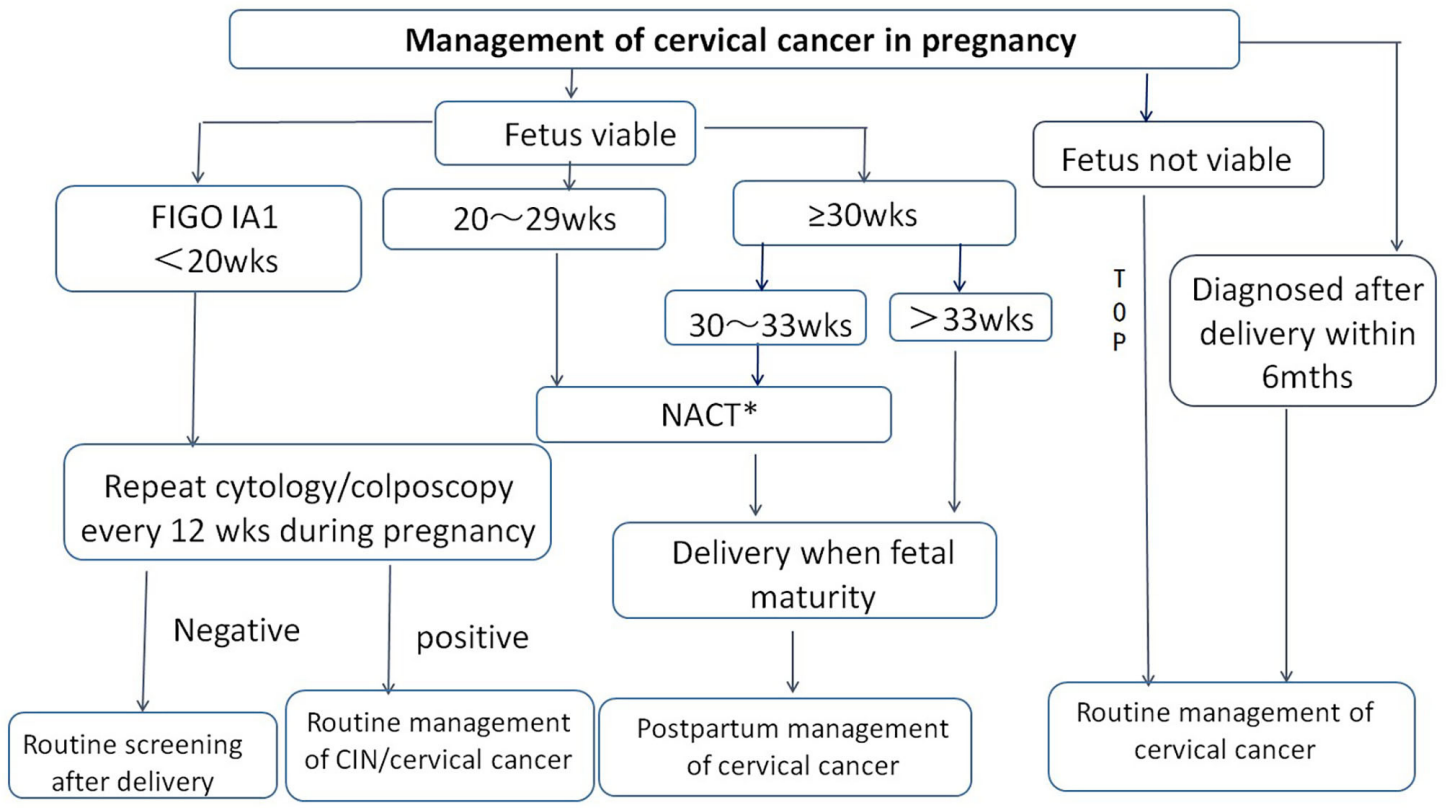
Management of cervical cancer in pregnancy by CSCCP recommendation. TOP, termination of pregnancy; NACT, neoadjuvant chemotherapy; wks, weeks; mths, months. *For cervical cancer in pregnancy in stage IB1 and above, NACT may used as a treatment option to allow for fetal maturation during the second and third trimesters of pregnancy.

Given the epidemic and treatment status of CCIP in China, consensus on the management of CCIP was achieved by the Chinese Society for Colposcopy and Cervical Pathology (CSCCP) in 2018 (Figure 2). This document may serve as a reference for the next prospective study and to promote the establishment of more standardized treatment guidelines for CCIP.

### Limitations

Our study represents the largest Chinese trial of patients with CCIP, involving 17 hospitals from 12 provinces in northern, eastern, and central China. A weakness of our study is that 21 patients who had initially been included had no data on survival, which prevented us from obtaining an accurate estimate of maternal survival. Secondly, NACT was only applied in 10 cases in the COP group, which limited our power to demonstrate the efficacy and adverse effects of NACT. Furthermore, some infants were lost to follow-up, and the median follow-up was too short to provide mid- and long-term outcomes for infants. Adopting a retrospective design was inevitable to collect enough data for the outcome analyses. Consequently, this study design may have affected the descriptive statistics or survival analyses performed in the study cases.

## Conclusion

In conclusion, educating the public about the importance of cervical cancer screening and the HPV vaccine is essential, especially for those not being screened regularly before pregnancy. Administration of platinum-based NACT during the second and third trimesters is a safe and preferred option. When counseling patients on the treatment modalities available during pregnancy, it is important to consider that the oncologic outcome of patients who choose to continue a pregnancy is similar to those who choose to terminate the pregnancy.

## Data Availability Statement

All datasets generated for this study are included in the article/supplementary material.

## Ethics Statement

The studies involving human participants were reviewed and approved by Ethics committee of Peking University people's hospital. The patients/participants provided their written informed consent to participate in this study.

## Author Contributions

ML and LW conceived and designed the study. ML, YZhao, MQ, YZhan, LL, BL, RG, ZY, RA, JL, ZZ, HB, YH, SC, GH, KH, QZ, QL, YW, and JW collected and analyzed the data. ML and XL prepared the manuscript. LW and XL edited the manuscript. All authors contributed to data analysis, drafting and revising the article, gave final approval of the version to be published, and agree to be accountable for all aspects of the work.

## Conflict of Interest

The authors declare that the research was conducted in the absence of any commercial or financial relationships that could be construed as a potential conflict of interest.
